# Supplementary material for: Plant and animal functional diversity drive mutualistic network assembly across an elevational gradient
Source: Nat Commun. 2018 Aug 9;9:3177. doi: 10.1038/s41467-018-05610-w (PMC6085337; doi:10.1038/s41467-018-05610-w)
Supplement: Supplementary file 1 — Supplementary Information [file 41467_2018_5610_MOESM1_ESM.pdf]

Supplementary Information

Plant and animal functional diversity drive mutualistic network assembly across an elevational gradient

Albrecht et al.

Supplementary Note 1

Jags model code. Monitored variables (highlighted in red font) were *mB*, a matrix of standardized effect sizes of the explanatory variables; *mP*, a selection matrix indicating whether an explanatory variable has been selected or not; and *rSq*, marginal and conditional variance explained by the models. Comments (#) are highlighted in blue font.

```
model {
  for (k in 1:nP) {# k = structural equation models [1:nP]
    ##########################################################################
    # Indicator model selection with random effect following O'Hara & Sillanpää[1]
    # - global shrinkage parameter tauB is estimated by the model
    # - the prior on the inclusion probability (gamma) is fixed at 0.5
    ##########################################################################
    # Priors
    sigB[k] ~ dunif(0, 20)
    tauB[k] <- pow(sigB[k], -2)
    for (j in 1:nY) {# j = response variables (Y) [1:nY]
      ##### fixed effects
      # intercepts
      betaT[1, j, k] ~ dnorm(0, 1e-4)
      gamma[1, j, k] <- 1
      beta[1, j, k] <- gamma[1, j, k] * betaT[1, j, k]
      # coefficients for main predictors
      for (x in 2:(nX[j] + 1)) {# x = predictor variables (X) [1:nX]
        gamma[x, j, k] ~ dbern(0.5)
        betaT[x, j, k] ~ dnorm(0, tauB[k])
        beta[x, j, k] <- gamma[x, j, k] * betaT[x, j, k]
      }
      ##### random effects
      for (r in 1:nRE) {# r = random effects (RE) [1:nRE]
        tau[r, j, k] <- pow(sig[r, j, k], -2)
        sig[r, j, k] ~ dunif(0, 20)
      }
      for (s in 1:nSite) {# s = random effect levels for site (siteRE) [1:nSite]
        siteRE[s, j, k] ~ dnorm(0, tau[1, j, k])
      }
      for (t in 1:nType) {# t = random effect levels for type (typeRE) [1:nType]
        typeRE[t, j, k] ~ dnorm(0, tau[2, j, k])
      }
      ##### Residual variance
      tau[3, j, k] <- 1/psiStar[j, j, k]
      ##### Explained variance
      # 1.) Variance explained by fixed effects
      # 2.) Variance explained by random effects
      rSq[1, j, k] <- pow(sd(mum[, j, k]), 2) /
        (pow(sd(mum[, j, k]), 2) + sum(pow(tau[, j, k], -1)))
      rSq[2, j, k] <- sum(pow(tau[1:nRE, j, k], -1)) /
        (pow(sd(mum[, j, k]), 2) + sum(pow(tau[, j, k], -1)))
      for (i in 1:nObs) {# i = observations [1:nObs]
        # Linear regressions
        mu[i, j, k] <-
          inprod(X[i, 1:(nX[j] + 1), k], beta[1:(nX[j] + 1), j, k]) +
          lambda[j, k] * eta[i, nE[j], k] +
          siteRE[RE[i, 1], j, k] + typeRE[RE[i, 2], j, k]
        # likelihood
        Y[i, j, k] ~ dnorm(mu[i, j, k], tau[3, j, k])
        # marginal predictions based on fixed effects
        mum[i, j, k] <-
          inprod(X[i, 1:(nX[j] + 1), k], beta[1:(nX[j] + 1), j, k]) +
          lambda[j, k] * eta[i, nE[j], k]
      }
    }
    ##### Latent variables for covariance terms
    for (i in 1:nObs) {
      eta[i, 1, k] ~ dnorm(0, 1)
      eta[i, 2, k] ~ dnorm(0, 1)
    }
    ##########################################################################
    # Parameter assignments & equality constraints for covariance terms
    # Code for implementing covariance terms is taken from R package 'blavaan'
    # (Merkle and Rosseel)[2]
    ##########################################################################
    lvRho[1, 2, k] <- -1 + 2 * covPars[5, k]
```

```
    lvRho[3, 4, k] <- -1 + 2 * covPars[6, k]
    psiStar[1, 1, k] <- psi[1, 1, k] - (sqrt(abs(lvRho[1, 2, k]) * psi[1, 1, k]))^2
    psiStar[2, 2, k] <- psi[2, 2, k] - ((-1 + 2 * step(lvRho[1, 2, k])) *
                                        sqrt(abs(lvRho[1, 2, k]) * psi[2, 2, k]))^2
    psiStar[3, 3, k] <- psi[3, 3, k] - (sqrt(abs(lvRho[3, 4, k]) * psi[3, 3, k]))^2
    psiStar[4, 4, k] <- psi[4, 4, k] - ((-1 + 2 * step(lvRho[3, 4, k])) *
                                        sqrt(abs(lvRho[3, 4, k]) * psi[4, 4, k]))^2
    lambda[1, k] <- sqrt(abs(lvRho[1, 2, k]) * psi[1, 1, k])
    lambda[2, k] <- (-1 + 2 * step(lvRho[1, 2, k])) *
      sqrt(abs(lvRho[1, 2, k]) * psi[2, 2, k])
    lambda[3, k] <- sqrt(abs(lvRho[3, 4, k]) * psi[3, 3, k])
    lambda[4, k] <- (-1 + 2 * step(lvRho[3, 4, k])) *
      sqrt(abs(lvRho[3, 4, k]) * psi[4, 4, k])
    # Inferential covariances
    psi[1, 2, k] <- lambda[1, k] * lambda[2, k]
    psi[3, 4, k] <- lambda[3, k] * lambda[4, k]
    # Priors
    for (j in 1:nY) {
      psi[j, j, k] <- pow(covPars[j, k], -1)
      covPars[j, k] ~ dgamma(1, .5)
    }
    covPars[5, k] ~ dbeta(1, 1)
    covPars[6, k] ~ dbeta(1, 1)
    ############################################################################
    # output for pathmodel
    ############################################################################
    # matrix with coefficients
    for (j in 1:nY) {
      mB[1:nX[j], j + 3, k] <- beta[2:(nX[j] + 1), j, k]
    }
    mB[5, 4, k] <- psi[1, 2, k]
    mB[4, 5, k] <- mB[5, 4, k]
    mB[6, 7, k] <- psi[3, 4, k]
    mB[7, 6, k] <- mB[6, 7, k]
    # matrix with selection probabilities
    for (j in 1:nY) {
      mP[1:nX[j], j + 3, k] <- gamma[2:(nX[j] + 1), j, k]
    }
    mP[5, 4, k] <- 1
    mP[4, 5, k] <- mP[5, 4, k]
    mP[6, 7, k] <- 1
    mP[7, 6, k] <- mP[6, 7, k]
  }
}
```

Supplementary Table 1. Correlation of plant and animal traits with the first and second RLQ axes.

Mutualism	Animal trait	*r*	*n*	*P*	Plant trait	*r*	*n*	*P*
Matching traits ~ RLQ axis 1; Moran's test: $P = 8.5 \times 10^{-5}$								
Bird–fruit	**bill width**	**-0.26**	**85**	**0.0004**	**fruit diameter**	**-0.26**	**63**	**0.0003**
Bird–flower	bill length	0.19	24	0.083	corolla depth	0.19	26	0.15
Insect–flower	**proboscis length**	**-0.21**	**183**	**0.0031**	**corolla depth**	**-0.16**	**131**	**0.026**
Energy traits ~ RLQ axis 1; Moran's test: $P = 1.8 \times 10^{-6}$								
Bird–fruit	**body mass**	**-0.24**	**85**	**0.0003**	crop mass	-0.11	63	0.078
Bird–flower	**body mass**	**-0.25**	**24**	**0.033**	**number of flowers**	**-0.23**	**26**	**0.048**
Insect–flower	**head width**	**-0.15**	**183**	**0.014**	**flowers per inflorescence**	**0.15**	**131**	**0.024**
Foraging traits ~ RLQ axis 1; Moran's test: $P = 0.031$								
Bird–fruit	kipp's index	-0.045	85	0.42	plant height	-0.10	63	0.20
Bird–flower	kipp's index	-0.10	24	0.39	**plant height**	**-0.27**	**26**	**0.018**
Insect–flower	**forewing index**	**0.13**	**183**	**0.012**	plant height	-0.033	131	0.66
Matching traits ~ RLQ axis 2; Moran's test: $P = 0.74$								
Bird–fruit	bill width	-0.043	85	0.53	fruit diameter	-0.067	63	0.26
Bird–flower	bill length	0.033	24	0.77	corolla depth	0.023	26	0.77
Insect–flower	proboscis length	0.029	183	0.72	corolla depth	0.016	131	0.84
Energy traits ~ RLQ axis 2; Moran's test: $P = 0.74$								
Bird–fruit	body mass	0.026	85	0.68	crop mass	0.10	63	0.079
Bird–flower	body mass	0.018	24	0.84	number of flowers	-0.0092	26	0.96
Insect–flower	head width	0.0094	183	0.90	flowers per inflorescence	0.028	131	0.72
Foraging traits ~ RLQ axis 2; Moran's test: $P = 0.23$								
Bird–fruit	**kipp's index**	**0.11**	**85**	**0.043**	plant height	0.071	63	0.38
Bird–flower	kipp's index	0.016	24	0.87	plant height	0.024	26	0.85
Insect–flower	forewing index	0.058	183	0.27	plant height	0.053	131	0.47

We used the fourth-corner permutation test (model 6 with 9,999 permutations)[3] to assess which of the different trait types (matching, energy and foraging traits) were correlated with each of the first two RLQ axes. As we aimed at generalizing our results across mutualisms, we mainly compared the absolute magnitude of correlations (Pearson's *r*) between matching, energy and foraging traits and the RLQ axes across the three mutualisms. In addition to the significance of individual correlations, we assessed the overall support for the hypotheses that matching, energy and foraging traits are related to the first and second RLQ axes across the three mutualisms. To do so, we used the equation given by Moran[4] (see Methods), based on a Bernoulli process, to calculate the probability, *P*, of obtaining a given number of significant tests from a given number of trials just by chance. The rationale behind this equation is that the evidence against the null hypothesis from a given number of statistical tests increases with the number of significant tests[4]. Previous work has shown that the sequential approach (permutation model 6) for statistical testing of the '*fourth-corner problem*' has good power (0.88) with large numbers of species (100), reasonable power (0.60) for 50 species and some power (0.40) for 30 species[5]. As the number of species in two of our datasets is relatively small, the statistical power of the permutation test is low. For instance, although the magnitude of the absolute correlations between matching traits and RLQ axis 1 in the bird–flower mutualism is similar to those in the bird–fruit and insect–flower mutualisms, the permutation test is not significant at $\alpha = 0.05$ (Supplementary Table 1; Supplementary Fig. 1). Therefore, the statistical tests we conducted can be considered highly conservative with respect to Type I errors. Correlations that are significant at $\alpha = 0.05$ are highlighted in boldface.

Supplementary Table 2. Summary of the stochastic variable selection in the Bayesian hierarchical structural equation models.

		(**a**) Niche Breadth ($ID = e^{H}$)					(**b**) Niche partitioning ($ID = d'$)				
X	Y	Effect (95% CI)	P	BF	PSRF	N_{eff}	Effect (95% CI)	P	BF	PSRF	N_{eff}
Effect of X on Y											
LU	FD_p	0 (-0.18, 0.11)	0.3	-1.7	1.001	4197	0 (-0.2, 0.11)	0.32	-1.5	1.003	3891
MAP	FD_p	**0.19 (0, 0.47)**	**0.77**	**2.4**	**1.001**	**3875**	**0.19 (0, 0.47)**	**0.78**	**2.5**	**1.003**	**4237**
MAT	FD_p	0 (-0.22, 0.17)	0.31	-1.6	1.003	4175	0 (-0.21, 0.16)	0.33	-1.4	1.003	4336
LU	FD_a	0 (-0.064, 0.23)	0.31	-1.6	1.001	4067	0 (-0.06, 0.24)	0.35	-1.3	1.004	4147
MAP	FD_a	0 (-0.12, 0.24)	0.34	-1.3	1.007	4231	0 (-0.12, 0.24)	0.35	-1.3	1.001	4435
MAT	FD_a	**0.2 (0, 0.43)**	**0.81**	**2.9**	**1.002**	**4042**	**0.19 (0, 0.42)**	**0.81**	**2.9**	**1.002**	**4215**
LU	ID_p	0 (-0.23, 0.042)	0.34	-1.3	1.002	4025	0 (-0.037, 0.19)	0.33	-1.4	1.003	3796
MAP	ID_p	0 (-0.16, 0.14)	0.29	-1.8	1.002	3929	0 (-0.11, 0.16)	0.29	-1.8	1.001	4124
MAT	ID_p	0.086 (0, 0.33)	0.62	0.94	1.003	4000	**0.37 (0.2, 0.55)**	**1**	**13**	1.003	3816
FD_p	ID_p	0 (-0.044, 0.15)	0.24	-2.3	1.003	3879	0 (-0.22, 0.013)	0.39	-0.88	1.001	3978
FD_a	ID_p	**0.4 (0.26, 0.54)**	**1**	**Inf**	**1.002**	**4554**	**0.25 (0.093, 0.39)**	**0.99**	**8.9**	**1.004**	**4103**
LU	ID_a	-0.011 (-0.31, 0.0077)	0.54	0.29	1.001	3874	0 (-0.12, 0.1)	0.25	-2.2	1.002	4160
MAP	ID_a	0 (-0.2, 0.11)	0.31	-1.6	1.002	3879	0 (-0.14, 0.15)	0.29	-1.8	1.002	4000
MAT	ID_a	**-0.27 (-0.47, 0)**	**0.95**	**5.9**	**1.003**	**3909**	**0.29 (0.045, 0.46)**	**0.98**	**7.7**	**1.004**	**3764**
FD_p	ID_a	**0.36 (0.2, 0.51)**	**1**	**17**	**1.002**	**4408**	**0.19 (0, 0.35)**	**0.92**	**4.8**	**1.000**	**4150**
FD_a	ID_a	0 (-0.22, 0.015)	0.39	-0.9	1.000	4090	0 (-0.18, 0.031)	0.32	-1.5	1.002	4289
Residual covariance											
FD_p	FD_a	0.19 (0.046, 0.36)	-	-	1.003	4337	0.19 (0.05, 0.36)	-	-	1.001	4384
ID_p	ID_a	0.11 (-0.0066, 0.24)	-	-	1.002	3945	0.26 (0.15, 0.41)	-	-	1.002	3783

Structural equation models for interaction niches of plants and animals (ID_p and ID_a) in terms of (**a**) niche breadth (partner diversity, e^{Hp} and e^{Ha}) and (**b**) niche partitioning (complementary specialization, d'_p and d'_a). LU, land use; MAP, mean annual precipitation, MAT, mean annual temperature; FD_p and FD_a, functional diversity of plants and animals (measured as functional dispersion). Given are pairs of predictor (X) and response (Y) variables along with effect sizes (with shrinkage) and 95% confidence intervals (CI), selection probabilities (P), $2\log_e$(Bayes factor) (BF), the potential scale reduction factor (PSRF) and effective sample size (N_{eff})[6]. Values of BF < 2 indicate no support; values between 2 and 6 indicate positive support; values between 6 and 10 indicate strong support; and values > 10 indicate decisive support. Paths with BF > 2 are highlighted in boldface type. Values of PSRF < 1.1 indicate that MCMC chains have converged on the same posterior distribution. N_{eff} indicates approximate sample size of posterior samples after accounting for temporal autocorrelation between posterior samples. Residual covariance terms were kept fixed during the model selection. Sample sizes are n_{obs} = 126 observations, n_{site} = 53 study sites and $n_{mutualism}$ = 3 mutualisms.

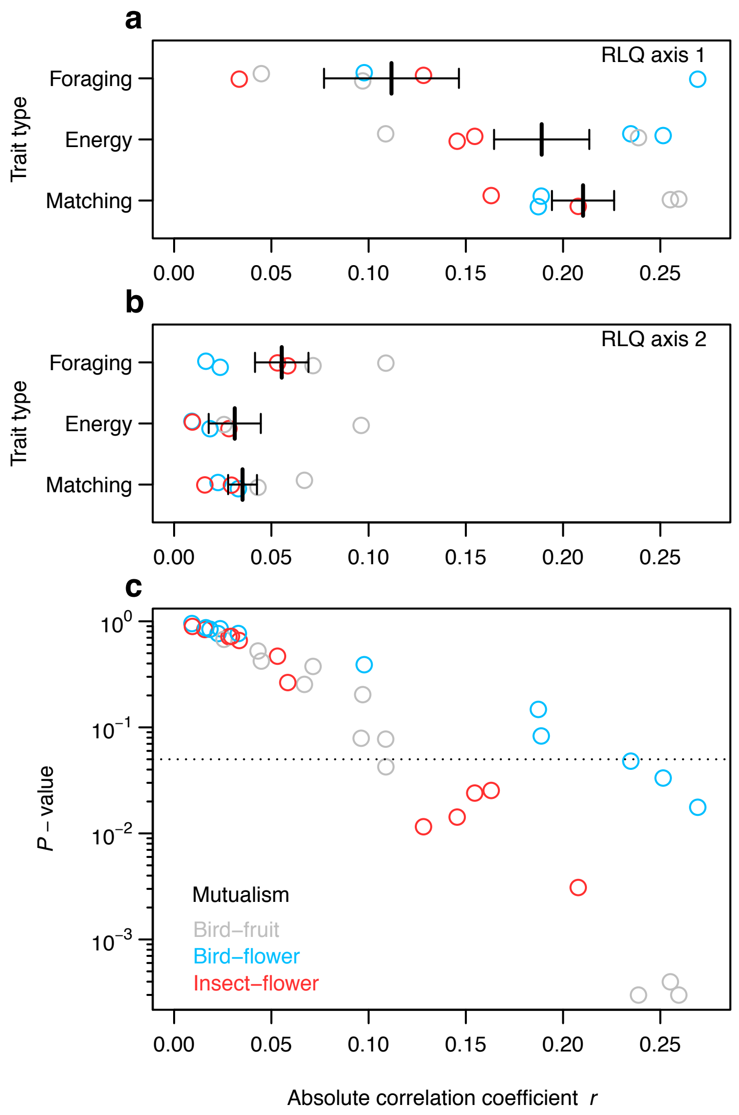

Supplementary Figure 1. Absolute correlation of different plant and animal trait types with RLQ axes in different types of plant–animal mutualistic networks. Absolute correlation coefficients (Pearson's *r*) of matching, energy and foraging traits with (**a**) the first RLQ axis and (**b**) the second RLQ axis. The first ordination axis explained most of the cross-covariance between plant and animal trait spaces (range across the three mutualisms: 90—99%), whereas the second axis explained only a minor proportion (range: 0.75—9.3%). **c** Relationship between the absolute correlation coefficient and the *P*-value of the permutation test in the fourth-corner analysis. Note that although the magnitude of the absolute correlations between matching traits and RLQ axis 1 in the bird–flower mutualism is similar to those in the bird–fruit and insect–flower mutualisms (**a**), the permutation test is not significant at α = 0.05 (dotted line in **c**). This is due to a lack of statistical power owing to the relatively small number of species in the bird–flower mutualism (26 plant and 24 bird species, respectively; see Supplementary Table 2)[5]. In **a**,**b** raw data (grey circles, bird–fruit; blue circles, bird–flower; red circles, insect–flower mutualism) and mean ± s.e.m. are shown. Note the logarithmic scale for the *y*-axis in **c**.

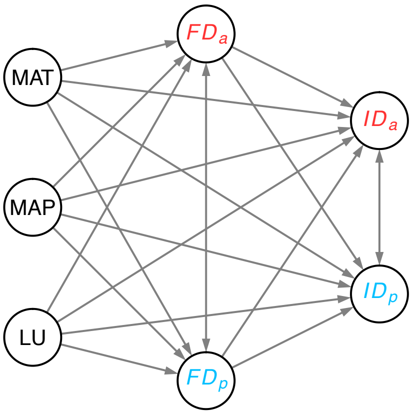

Supplementary Figure 2. Initial path model that was the basis for the variable selection. We fitted two separate structural equation models, one including e^{Hp} and e^{Ha} (partner diversity) and the other including d'_p and d'_a (complementary specialization) as measures of the interaction niches of plant and animal communities on each study site (represented by ID_p and ID_a in the model). In these structural equation models, we treated mean annual temperature (MAT, °C), mean annual precipitation (MAP, mm yr^{-1}) and land use (LU, binary variable) as exogenous predictor variables. We treated functional diversity (FD_p and FD_a) as well as metrics of interaction niches (ID_p and ID_a) as endogenous variables. The models included all potential direct effects of MAT, MAP and LU on FD_p and FD_a, as well as on ID_p and ID_a, respectively. Moreover, the models included the effects of FD_p and FD_a on ID_p and ID_a, respectively. We also included covariance terms between FD_p and FD_a, as well as between ID_p and ID_a to account for correlated errors due to common unmeasured sources of variance.

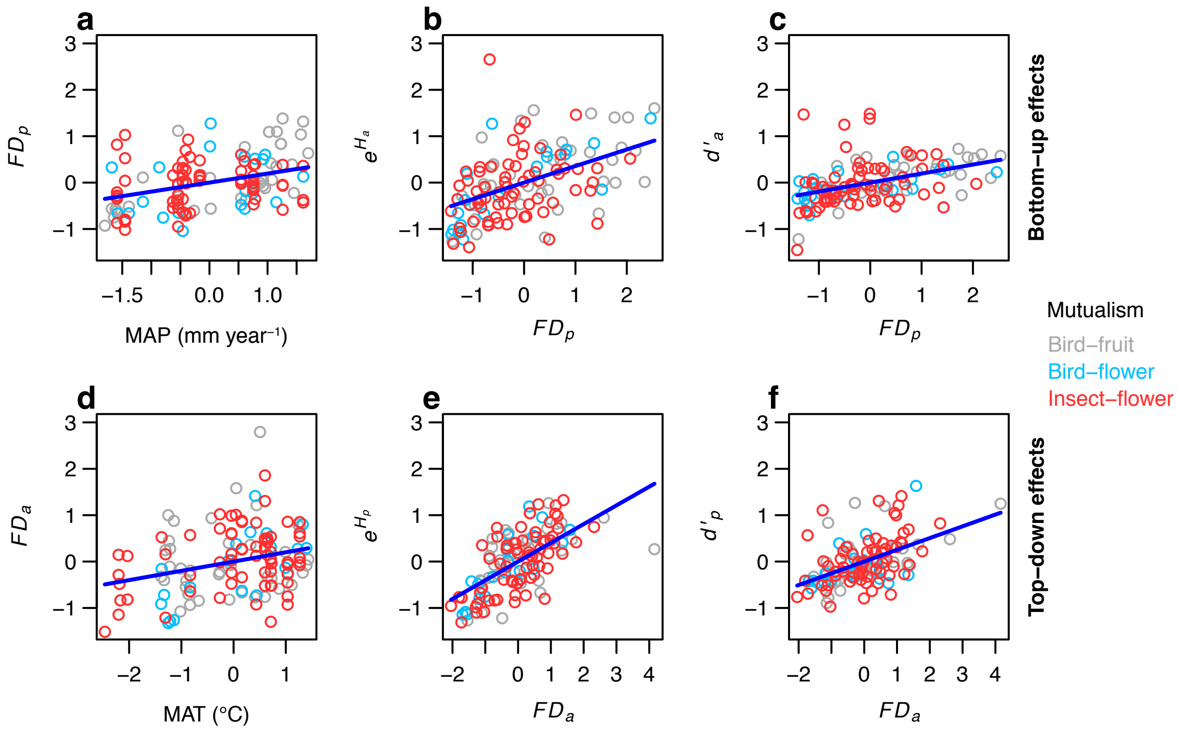

Supplementary Figure 3. Partial residual plots of the indirect '*functional diversity*'-mediated effects of abiotic factors on niche breadth and partitioning in plant–animal mutualistic networks. Plots **a**–**f** visualize partial relationships indicated by the path analysis in Fig. 3. **a** Effect of mean annual precipitation (MAP, mm yr^{-1}) on plant functional diversity (functional dispersion, FD_p). **b**,**c** Bottom-up effects of FD_p on niche breadth (partner diversity, e^{Ha}) and partitioning of animals (complementary specialization, d'_a). **d** Effect of mean annual temperature (MAT, °C) on animal functional diversity (FD_a). **e**,**f** Top-down effects of FD_a on niche breadth (e^{Hp}) and partitioning of plants (d'_p). All variables were scaled to zero mean and unit variance before analysis. Units on the *y*-axes are standardized residual deviations from predicted partial scores after conditioning on all predictor variables except for the one shown on the *x*-axis and after conditioning on the two random effects (study site and mutualism type). The colours of the circles represent the three mutualisms (grey, bird–fruit; blue, bird–flower; red, insect–flower). The blue lines depict the partial regression slopes. Sample sizes are n_{obs} = 126 observations, n_{site} = 53 study sites and $n_{mutualism}$ = 3 mutualisms.

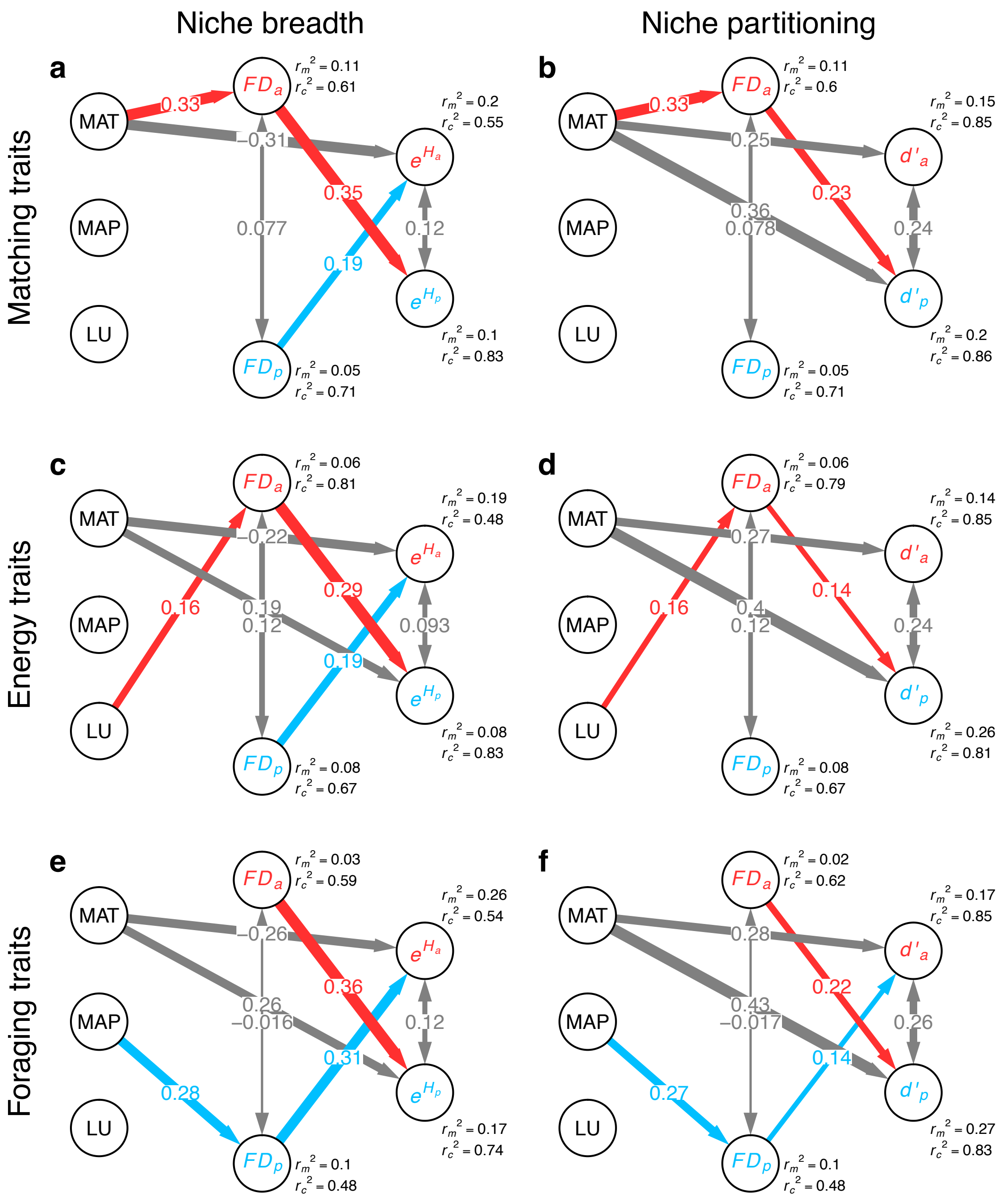

Supplementary Figure 4. Structural equation models testing for trait-specific bottom-up and top-down effects of functional diversity on the assembly of plant–animal mutualistic networks. The Bayesian hierarchical structural equation models in **a**-**f** tested for direct and indirect 'functional diversity'-mediated effects of mean annual temperature (MAT, °C), mean annual precipitation (MAP, mm yr^{-1}) and land use (LU, binary variable) on (**a**,**c**,**e**) niche breadth (partner diversity, e^H) and (**b**,**d**,**f**) niche partitioning (complementary specialization, d') of plants and animals in the mutualistic networks via functional diversity of plant and animal communities (functional dispersion, *FD*; subscripts *p* and *a* for plants and animals, respectively). The structural equation models are based on (**a**,**b**) matching traits, (**c**,**d**) energy traits or (**e**,**f**) foraging traits, respectively (see Methods). Only paths that were supported by the Bayesian variable selection (2log$_e$(Bayes factor) > 2) are shown. Path colours depict bottom-up mediated (blue), top-down mediated (red) and direct abiotic effects (grey) on network structure. Grey double-headed arrows depict covariance terms that account for correlated errors due to common unmeasured sources of variance and due to reciprocal effects of functional diversity in each trophic level on competitive exclusion in the other trophic level. Path widths are proportional to standardized effect sizes. The values near the endogenous variables depict the marginal (r_m^2) variance explained by fixed factors only and the conditional (r_c^2) variance explained by fixed and random factors combined (see Methods). Sample sizes are n_{obs} = 126 observations, n_{site} = 53 study sites and $n_{mutualism}$ = 3 mutualisms.

Supplementary References

1. O'Hara, R. B. & Sillanpää, M. J. A review of Bayesian variable selection methods: what, how and which. *Bayesian Analysis* **4,** 85–117 (2009).
2. Merkle, E. C. & Rosseel, Y. blavaan: Bayesian structural equation models via parameter expansion. *arXiv* (2015). doi:10.18637/jss.v085.i04
3. Dray, S. *et al.* Combining the fourth-corner and the RLQ methods for assessing trait responses to environmental variation. *Ecology* **95,** 14–21 (2014).
4. Moran, M. D. Arguments for rejecting the sequential Bonferroni in ecological studies. *Oikos* **100,** 403–405 (2003).
5. ter Braak, C. J. F., Cormont, A. & Dray, S. Improved testing of species traits–environment relationships in the fourth-corner problem. *Ecology* **93,** 1525–1526 (2012).
6. Plummer, M., Best, N., Cowles, K. & Vines, K. CODA: convergence diagnosis and output analysis for MCMC. *R News* **6,** 7–11 (2006).
